# Non-Thermal Atmospheric Pressure Plasma as an Adjunct to Intestinal Anastomosis: A Pilot Study on Preventing Anastomotic Leaks

**DOI:** 10.3390/life14111450

**Published:** 2024-11-08

**Authors:** Mario Betancourt-Ángeles, Régulo López-Callejas, Guillermo Berrones-Stringel, César Jaramillo-Martínez, Bryan Navarro-Luna, Benjamín Gonzalo Rodríguez-Méndez, Antonio Mercado-Cabrera, Raúl Valencia-Alvarado

**Affiliations:** 1 Medical Center ISSEMyM Toluca, Av. Baja Velocidad 284 km. 57.5, San Jerónimo Chicahualco, Metepec 52170, Mexico; mariobeta74@hotmail.com (M.B.-Á.); stringelb@hotmail.com (G.B.-S.); cesar_jara77@yahoo.com (C.J.-M.); bryanmd.5510@gmail.com (B.N.-L.); 2 Plasma Physics Laboratory, Instituto Nacional de Investigaciones Nucleares, Carretera México-Toluca S/N, La Marquesa, Ocoyoacac 52750, Mexico; regulo.lopez@inin.gob.mx (R.L.-C.); antonio.mercado@inin.gob.mx (A.M.-C.); raul.valencia@inin.gob.mx (R.V.-A.)

**Keywords:** intestinal anastomosis, leakage, non-thermal atmospheric pressure plasma

## Abstract

Anastomotic leaks remain a significant challenge in intestinal surgery, often leading to severe complications. This study investigated a novel approach to enhance anastomotic healing and reduce the risk of leaks by combining traditional suturing and stapling techniques with non-thermal atmospheric pressure plasma (NTAPP) application. NTAPP, a cold atmospheric plasma generated through the ionization of ambient air, has been shown to possess antimicrobial, hemostatic, and wound-healing properties. NTAPP promotes sterilization, coagulation, and tissue regeneration by generating reactive oxygen and nitrogen species, potentially strengthening anastomotic union. This pilot study evaluated the efficacy of NTAPP in three patients undergoing intestinal anastomosis. Following the standard surgical procedure, NTAPP was applied directly to the anastomotic site. Postoperative outcomes were monitored for six months, including anastomotic leaks and healing rates. Preliminary results demonstrated promising outcomes. All three patients exhibited successful sealing of the anastomosis, with no evidence of leakage during the follow-up period, providing reassurance and confidence in the potential of sutures, staples, and NTAPP. These findings suggest that NTAPP can significantly improve the safety and efficacy of intestinal surgeries by reducing the incidence of anastomotic leaks. While further research with a larger sample is necessary to confirm these initial findings, the results of this study provide a strong foundation for exploring the potential of NTAPP as a valuable adjunct to conventional surgical techniques for preventing anastomotic leaks. This innovative approach could reduce postoperative complications, improve patient outcomes, and enhance the overall quality of care in intestinal surgery.

## 1. Introduction

Intestinal anastomosis, a surgical procedure involving the joining of two intestinal segments, is a cornerstone of gastrointestinal surgery. It is employed in treating various pathologies, such as inflammatory bowel disease [1], intestinal obstructions [2], and intestinal tumors [3]. This process aims to restore intestinal continuity [4] or divert intestinal transit to a new route.

Several factors influence the healing process of intestinal anastomoses, including patient comorbidities [5] and tissue quality. Primary healing by precise apposition is ideal [6], but most anastomotic techniques rely on secondary healing. Fortunately, the intestine’s abundant blood supply and intra-abdominal location facilitate healing [7].

During the delayed phase of intestinal anastomosis, surgeons play a crucial role in providing extrinsic support to maintain tissue continuity [3,8]. Given the limited resistance of intestinal anastomoses to longitudinal distension and distraction, this support is essential for optimal healing. However, anastomotic leakage (AL) remains a significant challenge after bowel surgery, potentially leading to peritonitis, prolonged hospital stays, and increased mortality [9]. The need for more specificity in current diagnostic methods hinders early detection, negatively impacting patient outcomes. This issue is urgent, and researchers are actively seeking diagnostic or predictive biomarkers of anastomotic leaks to improve diagnostic accuracy and clinical management. Proper healing of intestinal anastomosis is essential to prevent postoperative complications [10]. Sutures, staples, and tissue adhesives are crucial in joining the intestinal segments. While absorbable sutures are preferred, staples and tissue adhesives offer alternative options.

Innovative methods for anastomotic closure have been developed to improve efficiency and reduce complications [11]. These include advanced technologies such as automated suturing devices [12] and improved stapling systems [13]. Double-stapled anastomoses [14] and the Kono-S stapled configuration [15] have been explored to reduce the risk of leaks. Additionally, tissue adhesive application techniques [16] offer promising alternatives for strengthening the anastomotic junction and accelerating healing.

Laser use in resolving leaks in intestinal anastomosis has gained interest due to its potential for fast, efficient, and automated joining [17,18]. Despite system complexity and parameter optimization challenges, laser intestinal joining has shown promise in improving union strength and reducing inflammation [19,20]. While laser welding with sutures has shown promising results, further research is needed to address challenges such as creating uniform unions on irregular surfaces and optimizing parameters [21]. With continued research, laser welding may be valuable in gastrointestinal surgeries.

Angiogenesis, crucial for wound healing, is often inhibited in adults. Fibroblasts play a crucial role in wound healing, migrating under the influence of vascular endothelial growth factor (VEGF), essential for angiogenesis, collagen deposition, and epithelialization [22]. While targeting VEGF is essential, it presents challenges in wound healing [23]. NTAPP has shown promise in generating VEGF and stimulating angiogenesis [24].

Non-thermal atmospheric pressure plasma (NTAPP) has emerged as a promising tool for promoting wound healing, as demonstrated by our previous studies in animal and human models [25,26,27,28,29] and others in the literature [24,30,31,32]. However, its application in intestinal surgery is a relatively unexplored field. This study aimed to evaluate the potential of NTAPP to improve intestinal surgery outcomes, focusing on anastomosis. Specifically, we sought to determine whether NTAPP can stimulate cell proliferation, reduce the risk of complications, and improve the safety of intestinal anastomoses.

## 2. Materials and Methods

### 2.1. NTAPP-Generation System

**Figure 1:**
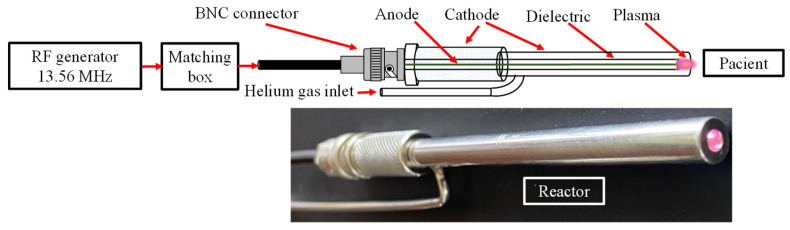
NTAPP generator schematic.

The NTAPP-generation system (designed and built in our laboratory), illustrated in Figure 1, comprised a copper filament (Condumex, Mexico City, Mexico) treated with a glow discharge plasma to enhance its biocompatibility, serving as the central electrode (anode). This electrode was enclosed within a ceramic tube (CoorsTek Inc., Golden, CO, USA), a crucial electrical insulation component. The ceramic tube was housed within a stainless-steel tube (Swagelok, Solon, OH, USA) that acted as the cathode. These electrodes were powered by an RF generator (Kurt J. Lesker Co., Pittsburgh, PA, USA) operating at a frequency of 13.56 MHz with a fixed power of 20 W, transmitted through a coaxial cable with a BNC connector (RENHOTEC, Jiangmen, China).

Helium (Criogas, S.A. de C.V., Toluca, Mexico) as working gas was supplied between the dielectric and the external electrode through a constant flow regulator (Mada Inc., Carlstadt, NJ, USA) of 0.5 L/min using a stainless-steel tube (Swagelok, Solon, OH, USA). This gas served as a medium for the plasma generation process. The configuration generated NTAPP with an irradiance of 0.45 W/cm^2^, which was applied to the anastomosis. These parameters aligned with the guidelines established by the International Commission on Non-Ionizing Radiation Protection [33], ensuring safe operation.

The reactor outlet nozzle was designed with an internal diameter of 3 mm, optimizing plasma flow while maintaining a temperature below 30 °C to safeguard patient tissue from thermal damage. Grounding the plasma reactor cathode and RF-generating equipment minimized the risk of arc flash, further enhancing patient safety.

A rigorous equipment preparation protocol was followed to ensure patient safety and the procedure’s effectiveness. Reactors were sterilized at the Sterilization and Equipment Center (SEC), cables were protected with sterile stockings, and the RF generator underwent bimonthly preventive maintenance.

### 2.2. Application Protocol

NTAPP was meticulously applied to the anastomosis for 1 min per centimeter, a duration optimized based on extensive preliminary studies [28,29]. This application time balanced the generation of reactive oxygen and nitrogen species, essential for wound healing and disinfection, with the potential for thermal effects and tissue damage. Patient safety was a primary concern, and potential side effects were carefully monitored. The optimized application time ensured an effective and safe treatment for intestinal anastomosis, providing a solid foundation for our research.

### 2.3. Safety Considerations

Rigorous safety protocols were implemented throughout the study to ensure patient well-being. The custom-designed NTAPP needle reactor incorporated advanced safety features, including precise control of gas flow, real-time temperature monitoring, and practical grounding. These measures were essential to maintain the integrity of patient tissues and prevent unintended thermal damage or electrical hazards. The plasma parameters, such as voltage and frequency, were carefully calibrated, balancing between optimizing therapeutic efficacy and minimizing the risk of adverse events. Moreover, the treatment area was continuously and diligently monitored for any signs of tissue damage or adverse reactions, ensuring patient safety at all times. These comprehensive safety measures underscore the commitment to patient-centered care and contribute to the overall robustness of the study.

### 2.4. Patients

The study adhered to the ethical principles outlined in the Declaration of Helsinki. The medical protocol was reviewed and approved by the ISSEMyM Medical Center Research Ethics and Health Research committees and classified as minimal risk. Participants were selected according to the registered medical protocol UEeIM 091/22.

Patients over 18 years of age with a diagnosis of catastrophic abdomen, whether admitted to the emergency department or hospitalized, were included, including those with disorders affecting wound healing, provided they were hemodynamically stable, regardless of body mass index or physical status. All participants signed the informed consent form to express their willingness to participate in the study. Those with psychiatric disorders, a history of seizures, or who were taking medications known to affect the central nervous system, as well as those who refused to participate in the study, were excluded.

### 2.5. Patient Characteristics

Patient 1: A 78-year-old male, a resilient individual with a 30-year history of Crohn’s disease characterized by recurrent exacerbations, including abdominal pain, diarrhea, and weight loss. The patient had undergone multiple surgical interventions, including a small-bowel resection and an ileostomy. He had also received a variety of immunosuppressive medications, including corticosteroids, azathioprine, and infliximab.

Patient 2: A 49-year-old male with a 15-year history of Crohn’s disease characterized by recurrent exacerbations, including abdominal pain, diarrhea, and weight loss. The patient, a symbol of perseverance, faced his condition complicated by hyperuricemia, which contributed to his overall symptoms. Despite treatment with medications, the patient’s symptoms continued to worsen, ultimately leading to intestinal obstruction secondary to ileocecal stenosis.

Patient 3: A 78-year-old female with a severe phenotype of Crohn’s disease, a case of utmost severity characterized by multiple comorbidities, including severe protein-calorie malnutrition, stage III chronic kidney disease, rheumatic heart disease, glaucoma, and major depressive disorder. The patient’s case was particularly severe, necessitating immunosuppressive therapy with benralizumab and admission for an intestinal restitution protocol due to the severity of her nutritional status and the presence of multiple comorbidities.

## 3. Results

In the surgeries, the plasma reactor depicted in Figure 1, which generates NTAPP by introducing helium as a working gas, was used. At the outlet nozzle, a visible plasma effluent is generated. Spectral analysis derived from the instrumentation detailed in Section 2.1 revealsed the presence of oxygen-based reactive species such as hydroxyl radical (•OH), singlet oxygen (O_2_(a^1^Δg)) and hydrogen peroxide (H_2_O_2_), the last identified by absorbance [34]. Nitrogen-based reactive species such as the γ-phase band of nitric oxide (NO), nitrogen oxide (NO_2_), and molecular nitrogen (N_2_^+^) were also observed [35]. The presence of reactive oxygen and nitrogen species, previously documented in our research [28,29], presents a double advantage by sterilizing the wound and simultaneously promoting healing, offering hope for improved surgical outcomes.

**Figure 2:**
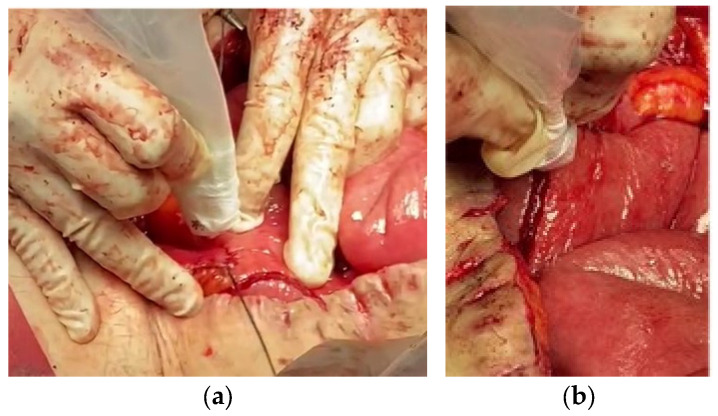
Application of NTAPP to the anastomosis line: (**a**) treatment over the reinforcing suture to improve the union’s security; (**b**) application over the stapled line to optimize healing and prevent complications.

In patient 1, a mechanical side-to-side jejunum colonic anastomosis was performed, overcoming grade II–III adhesions and resolving a mucous fistula. After suturing and stapling the area, these were treated with NTAPP to promote healing and prevent infection (Figure 2). Jackson–Pratt drains were placed to manage postoperative exudate. The patient tolerated the procedure well and showed no signs of infection at the 180-day follow-up. The anastomosis healed without evidence of dehiscence or stenosis. These preliminary results suggest that applying NTAPP in combination with conventional surgical techniques may improve postoperative outcomes in patients with Crohn’s disease and complex surgical complications by reducing the risk of infectious complications and promoting faster healing while maintaining high safety and patient comfort.

**Figure 3:**
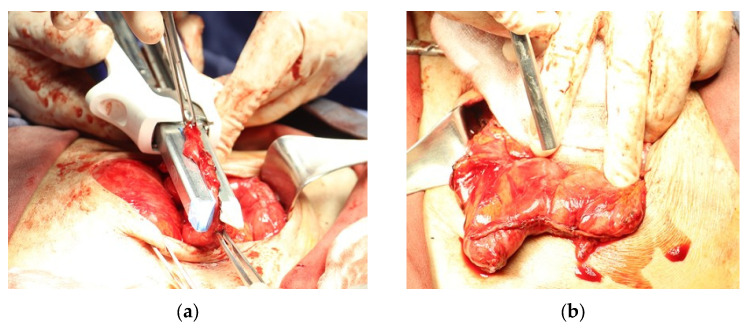
Surgical procedure. (**a**) Formation of the anastomosis by stapling. (**b**) Direct application of NTAPP effluent on the suture line and stapling.

For patient 2, a 25 cm-long circumferential ileocecal stricture was confirmed during surgery. A segmental resection was performed with utmost precision, and a mechanical suture-assisted side-to-side entero-enteric anastomosis was created using a 60 mm automatic linear stapler. Due to tension in the anastomotic line, it was decided to reinforce the union with additional sutures. The advanced technique of NTAPP (Figure 3) was applied to promote hemostasis and to stimulate healing through the release of reactive oxygen species, ensuring the best possible outcome for the patient. To prevent complications, a Jackson–Pratt drain was placed in the pelvic cavity. Estimated blood loss was 50 cc. The patient tolerated the procedure well and was transferred to the postoperative care unit, where he received antibiotic prophylaxis and a liquid diet and was mobilized early. After 48 h, the patient had good intestinal transit and no fever. The patient was followed up for 180 days, and the anastomosis healed without evidence of dehiscence or stenosis.

**Figure 4:**
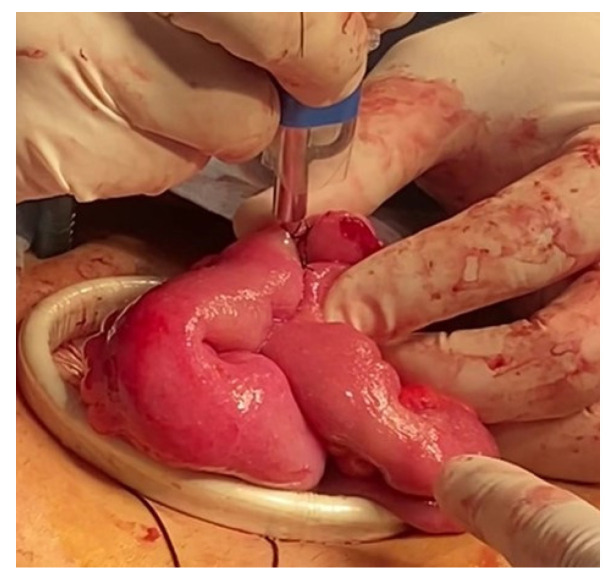
Optimization of intestinal anastomosis healing using NTAPP in laparoscopic Meckel diverticulum surgery.

Finally, in patient 3, during the surgical intervention, a laparoscopic approach with four trocars was performed. Initially, a supra-infraumbilical approach in the paramedian line introduced the optics and instruments. A significant presence of inter-loop and loop–wall adhesions was observed, which were released using adherenciolysis with advanced bipolar energy, restoring the mobility of the affected organs and minimizing the risk of future complications. A Meckel’s diverticulum was confirmed, with detection two small peritoneal cysts and mucous fluid on palpation of its distal portion, suggestive of inflammation or infection. Given this situation, laparoscopic resection of the Meckel’s diverticulum was performed. To perform the end-to-end enter-enteric anastomosis, the intestinal ends were exteriorized through a supra-infraumbilical incision on the paramedian line created for the laparoscopic procedure. The anastomosis was performed using a mechanical stapler for initial joining and subsequently reinforced with a running suture. To optimize healing and prevent complications, NTAPP was applied over the anastomosis line (Figure 4). Considering the thorough intraoperative evaluation, which included verification of hemostasis and anastomotic integrity by hydraulic testing and direct observation of excellent suture line healing, it was decided to omit drain placement. These evaluations supported confidence in the strength of the anastomosis, minimizing the risk of postoperative complications.

The decision to omit the Jackson–Pratt drain in this patient was based on a thorough intraoperative evaluation. After NTAPP application and integrity testing, the anastomosis was found to be solid, and together with the absence of active bleeding and adequate tissue tension, ruled out the risk of leaks. Postoperative progress was favorable, with high tolerance to the procedure and discharge from the hospital on the second day. After 180 days, satisfactory anastomosis healing was verified, with no evidence of dehiscence or stenosis.

Table 1 presents a detailed analysis of the intraoperative and postoperative clinical data of the three patients who underwent anastomotic surgery and were treated with NTAPP. Variables such as surgical time, blood loss, and length of hospital stay are included.

All three patients experienced successful healing of the intestinal anastomosis without evidence of dehiscence, stenosis, or infectious complications. The application of NTAPP in conjunction with conventional surgical techniques demonstrated promising results in improving postoperative outcomes, particularly in patients with complex surgical histories and multiple comorbidities. These findings, which reiterate the successful outcomes, suggest that NTAPP may be a valuable adjunct to intestinal anastomosis, reducing the risk of complications and enhancing patient recovery, thereby reassuring the audience about the approach’s effectiveness.

## 4. Discussion

In this study, a needle plasma reactor using helium as the working gas was characterized. The results demonstrate that this system generates a plasma rich in reactive species, with a significant increase in the production of hydroxyl radicals compared to our previous studies [28,29], which we attribute to the pointed geometry of the central electrode. This high concentration of hydroxyl radicals suggests more significant potential for sterilization and promotion of healing. The findings support the hypothesis that the NTAPP generated by our reactor can be an effective tool for intestinal anastomosis by promoting disinfection, angiogenesis, and modulation of the inflammatory response [24,36].

Intestinal anastomosis remains a challenging surgical procedure with a high risk of complications, such as leaks, bleeding, and stenosis [37]. These complications are often attributed to the introduction of foreign materials and the subsequent inflammatory response [38,39]. Factors such as suture material and tissue thickness influence the strength of the anastomosis [18].

Various surgical techniques have been developed to minimize complications [40,41]. However, anastomotic leakage remains a significant clinical problem, leading to peritonitis, prolonged hospital stays, and increased costs [42]. Factors contributing to anastomotic leakage include patient characteristics and technical factors related to the anastomosis [43].

In this context, using non-thermal plasma as an adjuvant in post-anastomotic wound healing is a promising alternative. By generating reactive oxygen and nitrogen species, non-thermal plasma can promote wound disinfection, stimulate angiogenesis, and modulate the inflammatory response, improving the integrity of the tissue junction and reducing the risk of leaks. Our results demonstrate that the plasma needle reactor used in this study accelerates the healing process and strengthens the anastomosis.

Traditionally, intestinal perfusion assessment is performed by subjective observations of color, pulsations, and bleeding. Near-infrared (NIR) fluorescence with indocyanine green (ICG) has revolutionized this assessment, providing an objective and real-time tool [44]. However, tissue perfusion is a complex process influenced by multiple factors. Using non-thermal plasma is a promising strategy to improve perfusion and wound healing in this context. By generating reactive oxygen and nitrogen species, non-thermal plasma stimulates angiogenesis, improves microcirculation, and modulates the inflammatory response, creating a favorable microenvironment for tissue repair. The combination of non-thermal plasma and NIR fluorescence could offer a more complete and accurate perfusion assessment, allowing early detection of potential complications and treatment optimization.

Sutureless techniques such as laser have shown potential in intestinal anastomosis, but are limited by the risk of thermal damage [20,45]. NTAPP, on the other hand, not only offers a promising alternative but also holds the potential to revolutionize the field by promoting healing and minimizing tissue damage [24]. While laser studies have demonstrated precision and control [18,46], their long-term efficacy and safety require further clinical evaluation [13].

NTAPP strengthens sutures and reduces inflammation, improving anastomotic integrity and significantly reducing the risk of complications [24,47]. This leads to better patient outcomes and improved quality of life. Therefore, NTAPP represents a significant advance in intestinal anastomosis surgery, offering a promising alternative to traditional techniques.

Mechanical tension determines the etiology of anastomotic leaks, compromising tissue integrity and junction healing. Excessive tension induces cell damage, ischemia, and hematoma, weakening the anastomosis. Recent studies [48,49] underline optimal tension’s importance in promoting healing and preventing complications. Atraumatic surgical techniques, biocompatible suture materials, and meticulous postoperative management are recommended to minimize tension. NTAPP emerges as a promising therapeutic strategy to address this problem. Existing evidence suggests that NTAPP stimulates angiogenesis, promotes cell proliferation, and modulates the inflammatory response, essential factors for tissue repair. Furthermore, by increasing the synthesis of collagen and extracellular matrix components [24,50], NTAPP can strengthen the mechanical strength of the anastomosis. Although further studies are required to establish its efficacy and safety conclusively, preliminary data suggest that NTAPP could be a valuable tool to optimize anastomotic healing and reduce the risk of leaks, especially in high mechanical stress.

The relationship between NSAID use and the incidence of anastomotic leaks is a topic of growing interest [51,52], although further research is still required to establish a definitive association. NTAPP emerges as a promising technology to address this problem, thanks to its ability to accelerate healing and reduce inflammation. In the context of patients with ileostomy, while this intervention may improve quality of life, its impact on the response to NTAPP is not yet fully elucidated [53,54,55]. Further studies are needed to determine the efficacy and safety of NTAPP in this subgroup of patients, as well as to assess its potential to mitigate the risks associated with both the use of NSAIDs and the presence of an ileostomy.

Several studies [56,57,58] suggest that late anastomotic leaks in patients undergoing sphincter-preserving surgery after neoadjuvant chemoradiotherapy are associated with a chronic inflammatory response characterized by a lower neutrophil-to-lymphocyte ratio. This chronic inflammation could contribute to the weakening of the anastomosis and increase the risk of leaks. Given the ability of NTAPP to modulate inflammation and promote healing, its application in this clinical context could represent an innovative and effective therapeutic strategy to prevent postoperative complications.

Although the costs of intestinal anastomosis treatments may vary [59,60], NTAPP represents an economically viable option. Its low cost per minute of application and its potential to reduce complications and decrease hospital stays suggest an excellent cost–benefit ratio, making it an attractive tool for optimizing the management of anastomotic leaks.

## 5. Conclusions

The results of this study demonstrate that NTAPP is a promising tool for optimizing wound healing and preventing complications in patients with Crohn’s disease undergoing intestinal surgery. The application of NTAPP, in combination with mechanical staplers and sutures, significantly accelerated the healing process and reduced the risk of postoperative infections, as indicated by the findings from the monitoring of Jackson–Pratt drains. These results support the hypothesis that NTAPP enhances the integrity of sutures and staples, creating a favorable wound-healing environment and minimizing the risk of dehiscence. Moreover, integrating these surgical techniques improved the consistency in performing anastomoses. In conclusion, NTAPP appears to be a valuable adjunct in the surgical management of patients with Crohn’s disease, enhancing clinical outcomes and the quality of life of patients. However, the need for large-scale studies to validate these findings and establish standardized clinical protocols for applying NTAPP in gastrointestinal surgery is paramount, underscoring the importance of further research in the field.

## Figures and Tables

**Table 1:** Clinical data of patients who underwent anastomotic surgery and were treated with NTAPP.

Parameter	Patient 1	Patient 2	Patient 3
Age [years]	78	49	78
Sex	Male	Female	Female
Surgery type	Partial colectomy	Ileocecal segmental resection	Laparoscopic resection of Meckel’s diverticulum
Anastomosis type	Terminal Term	Laterolateral entero-enteric	Terminoterminal entero-enteric
Surgical technique	Techniques combination	Techniques combination	Laparoscopic using four trocars
Stapler	linear	linear	linear
Suture material	Polyglactin 910 (Vicryl)	Polyglactin 910 (Vicryl)	Polyglactin 910 (Vicryl)
Suture gauge	2-0	2-0	2-0
Number of planes	2 (stapler and manual)	2 (stapler and manual)	2 (stapler and manual)
Irrigation	saline solution	saline solution	saline solution
Drain	Jackson-Pratt	Jackson-Pratt	Not placed
Surgical time	120 min	135 min	100 min
Blood loss	200 mL	50 mL	25 mL
Intraoperative complications	Neither	Neither	Adhesions, Meckel’s diverticulum
Postoperative complications	None mentioned	None mentioned	None mentioned
Hospital stay time	7 days	7 days	3 days
Epithelialization	Complete	Complete	Complete
Neovascularization	Observed	Observed	Observed
Inflammation	slight	slight	very slight
Fibrosis	It is observed	It is observed	It is observed
Signs of infection	Not found	Not found	Not found
Dehiscence	Not	Not	Not
Stenosis	Not	Not	Not
Necrosis	Not	Not	Not

## Data Availability

We regret to inform that due to ethical restrictions, we cannot provide specific clinical data for the article.
